# Effective Interactions between Double-Stranded DNA Molecules in Aqueous Electrolyte Solutions: Effects of Molecular Architecture and Counterion Valency

**DOI:** 10.1021/acs.jpcb.3c02216

**Published:** 2023-07-26

**Authors:** Terpsichori S. Alexiou, Christos N. Likos

**Affiliations:** Faculty of Physics, University of Vienna, Boltzmanngasse 5, 1090 Vienna, Austria

## Abstract

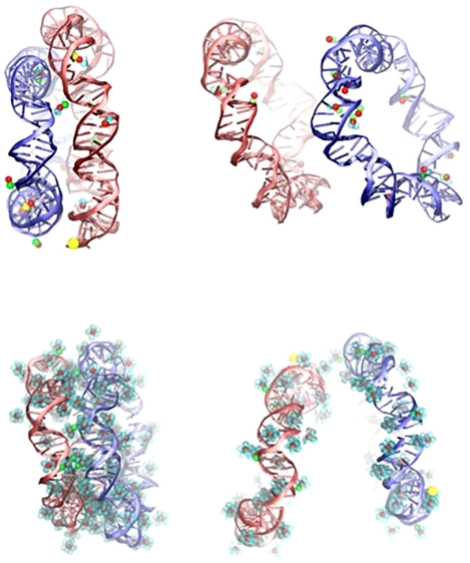

A computational investigation of the effects of molecular topology, namely, linear and circular, as well as counterion valency, on the ensuing pairwise effective interactions between DNA molecules in an unlinked state is presented. Umbrella sampling simulations have been performed through the introduction of bias potential along a reaction coordinate defined as the distance between the centers-of-mass of pairs of DNA molecules, and effective pair interaction potentials have been computed by employing the weighted histogram analysis method. An interesting comparison can be drawn between the different DNA topologies studied here, especially with regard to the contrasting effects of divalent counterions on the effective pair potentials: while DNA–DNA repulsion in short center-of-mass distances decreases significantly in the presence of divalent counterion-ions (as compared to monovalent ions) for linear DNA, the opposite effect occurs for the DNA minicircles. This can be attributed to the fact that linear DNA fragments can easily adopt relative orientations that minimize electrostatic and steric repulsions by rotating relative to one another and by exhibiting more pronounced bending due to the presence of free ends.

## 1 Introduction

In the crowded intracellular milieu, DNA molecules frequently assume circular molecular conformations, ranging from simple, topologically unlinked configurations (even exhibiting supercoiling or knots) to structurally complex, topologically interlinked configurations, such as the kinetoplast DNA (kDNA). Spontaneous self-assembly of DNA molecules is ubiquitous in biological systems, and DNA–DNA interactions are relevant to numerous biological processes, including genome compaction^1^ and homologous recombination, as well as in emerging DNA nanotechnological applications, such as functionalized DNA origami^2,3^ and synthesized DNA-catenanes.^4^

Tightly packaged DNA structures occur naturally in vivo, a typical example being nucleosome core particles, the storage unit of eukaryotic genomes, where approximately 150-bp-long double stranded (ds) DNA segments wrap around a histone complex protein core. Genomic DNA packaging in viral capsids yields toroidal, highly condensed DNA condensates that comprise multiple circumferentially wound and hexagonally packed DNA helices, resulting in DNA segments that are locally aligned, separated by just one or two layers of water.^5,6^ DNA condensation can be mediated in vitro by naturally occurring polyamines like spermidine (3+ charge) and spermine (4+ charge), yielding condensates that generally have a toroidal or rodlike shape. Interestingly enough, the resulting condensate size distribution appears to be nearly independent of the condensing agent used or even the DNA molecular length. DNA fragments with length ranging from 400 to 40,000 base pairs (bp) can invariably self-organize into toroids of approximately (invariantly constant) 100 nm external diameter, but shorter DNA molecules (e.g., 150-bp mononucleosomal DNA) cannot condense in this fashion.^6,7^

Considerable experimental and theoretical research effort has been devoted to the investigation of the intricate interplay among physicochemical parameters such as the DNA molecular length, the counterion valency and ionic strength, base sequence specificity, and degree of confinement, factors that appear to drive the various forms of self-organization of DNA in diverse biological settings. Self-organization of DNA in condensates, in particular, is enabled by a combination of individual effects, including the modification of the electrostatic interactions between DNA segments, fine-tuning of DNA–solvent interactions, volume exclusion, and localized helical bending or distortion.

The prominent role of counterions as chemical agents that drive DNA condensation and self-organization by screening the Coulombic repulsions between the DNA phosphates and by producing DNA segment attraction through correlated fluctuations of the ionic atmosphere has long been established, both experimentally and theoretically.^6,8−15^ At least 90% of the DNA charge needs to be neutralized by counterions so that condensation of long DNA can take place in aqueous solutions, and a valency of at least +3 is required. Cations of valency equal to +2 can condense supercoiled plasmids, although not in their torsionally relaxed or linearized form. A sufficiently high concentration of divalent ions can cause the condensation of linear DNA, but not in ordered condensates.^7^ Condensation is reversible upon a sustained salt concentration increase, and DNA condensates eventually redissolve due to the screening of short-range electrostatic attraction.^15^

The more efficient charge screening effected by divalent ions is readily reflected upon the values of the second virial coefficient, as obtained from static light scattering experiments of mononucleosomal DNA fragments (150–160 bp) in NaCl and CaCl_2_ solutions over a wide range of salt and DNA concentrations. More than 2-fold lower virial coefficient values were estimated in the presence of divalent calcium ions as compared to the respective values in sodium chloride solutions. Upon increasing ionic strength, the second virial coefficient values exhibit a marked decrease, signifying the Coulombic interaction screening.^10^ Similar qualitative trends have been observed with supercoiled, torsionally relaxed and linearized forms of the Col El plasmid DNA.^8^ Although a good qualitative agreement is observed against theoretical estimates of the charged rod model with respect to the ionic strength dependence, there is a marked quantitative discrepancy, and the second virial coefficient theoretical estimates consistently underestimate experimental observations.^10^ Good qualitative agreement with the charged rod model is observed mainly in the low salt regime for the case of aggregation of mononucleosomal DNA in NaCl. At a certain critical concentration which increases with added salt, the intensity of the equilibrium static scattering increases several 100-fold,^16^ indicating the DNA aggregates. The underlying mechanism cannot be sufficiently explained by theoretical models.

Even in the case of ultrashort ds linear DNA fragments as low as 8–25 bps, counterion mediated attraction can be induced by divalent magnesium counterions in sufficiently high concentration (16 mM Mg^2+^). Compared to sodium chloride solutions of the same ionic strength, inter-DNA repulsion is much weaker, and a shorter DNA length results in much stronger attraction.^17^ In a recent computational study,^18^ MD simulations were performed to quantify the effective potential of interaction between 30 bp long ds linear DNA fragments, periodically repeating along the longest axis dimension. DNA molecule pairs are bridged by sodium ions in both direct and water-mediated manner.

Although side-by-side attraction of short ds DNA fragments is more common, some studies suggest that an alternative, end-to-end stacking intermolecular interaction mechanism can also occur^19^ and becomes prevalent for ultrashort ds DNA. In the case that the local DNA structure deviates from the right-handed B-form, the most commonly occurring DNA structure in aqueous solutions, significant effects can be observed in the interaction between ds DNA fragments, as revealed by all-atom molecular dynamics (MD) simulations of the interactions between short (10 and 12 bp) fragments in the presence of MgCl_2_ electrolyte. For right-handed B-form DNA, weak attractive forces can be observed, while, on the contrary, for left-handed Z-DNA, significantly stronger attraction occurs, accompanied by a tight and stable bound state. A two-stage binding process of Z-DNA fragments, mediated by Mg^2+^, is outlined: two Z-DNA fragments can initially attract each other through charge screening, while at close enough distances, the divalent counterions form a hydrogen-bond network bridging intermolecular phosphates. Although tightly bound, minor-groove-to-minor locking DNA pair conformations have been observed in the presence of divalent magnesium ions for B-form DNA,^20^ the resulting attraction is still weaker compared to Z-DNA.

Overall, appreciable new insights into the underlying physics of DNA self-organization and the nature of the intermolecular forces have been revealed by experimental studies, through the application of novel techniques, the investigation of a wide range of condensing agents, and the application of recombinant DNA technology to produce fragments of desired length and defined sequence. Εffective attraction between DNA molecules in multivalent electrolytes has been clearly experimentally established. Several theoretical models have been proposed to unravel the origin of DNA attraction. Counterion correlation models, which approximate DNA as a uniformly charged cylinder and neglect discreteness of the DNA charge and other structural features, predict DNA condensation in an electrolyte for ions of valence greater than 3.^11^ The electrostatic zipper model accounts for the inhomogeneous charge distribution along DNA but assumes binding of counterions to the DNA grooves, which presumably renders them positively charged.^21^ In that model, effective attraction between two DNA molecules originates from interlocking positively charged grooves of one molecule with the negatively charged backbone of the other. However, common counterions found in biological cells, such as Na^+^, K^+^, Ca^2+^, and Mg^2+^ (valence <3), have high affinity to the DNA backbone, not to DNA grooves.

In the face of these shortcomings, simulations offer an appealing alternative route for the study of these systems at an atomistic level, allowing for the detailed observation of the microscopic molecular conformation of DNA and the ionic atmosphere.^22^ The scope of the work presented here is the computational investigation of the effects of DNA molecular architecture, namely, linear and circular, as well as counterion valency, on the ensuing pairwise effective interactions between DNA molecules in an unlinked state, within the dilute solution regime. The molecular lengths studied in this work are less than 200 bp and highly relevant to novel therapeutic applications of short DNA minicircles^23^ and linear DNA fragments.

The structure of the rest of the manuscript is as follows: In section 2, we provide details of the simulated DNA systems and the simulation protocol used to carry out biased umbrella sampling MD simulations. The potential of mean force along a single reaction coordinate, defined as the distance between the center-of-masses of the pairs of DNA molecules, is presented in section 3. Emphasis is placed on the investigation of the role of counterions and the mechanism of counterion condensation. Finally, in section 4, we summarize the main conclusions and outlook of the present work and outline future plans.

## 2 Systems Studied and Simulation Details

In this section, we present technical details regarding the simulated systems. Initial coordinates for duplex DNA structures were generated using the nab module of AmberTools.^24^ All generated structures were B-type DNA Arnott structures with a uniform rise of 3.38 Å and a twist uniformly distributed between each base pair step. To investigate the effect of molecular topology, a 24 bp linear duplex with sequence d(ATCG)6, and a 65 bp torsionally relaxed DNA minicircle with zero superhelical density and sequence
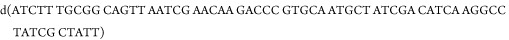
were constructed.

A parallel-oriented pair of either linear or minicircle DNA molecules was placed in a cubic simulation cell and solvated with the inclusion of SPC/E water molecules: typical initial configurations thereof are shown in Figure S1. Sufficiently large simulation cells were employed to avoid finite-size effects and to allow for the conduction of pulling (umbrella sampling) simulations along the *z*-axis. The aqueous solutions studied fall within the dilute concentration regime, with resulting DNA concentrations well below the corresponding overlap concentration limit for stiff rod molecules. Details of the properties of the systems investigated are provided in Table 1. For the neutralization of the DNA pair charges, an appropriate number of either sodium or calcium counterions was included, and then sufficient Na^+^Cl^–^ or Ca^2+^Cl^–^ ion pairs were added to achieve a bulk ionic strength of 0.1 M, using the Smith and Dang force field parameters^25^ for sodium and the Åpqvist force field parameters^26^ for calcium. All ions were initially placed randomly at distances at least 5 Å from the solute and at least 3.5 Å from one another. The last generation general-purpose AMBER force field that takes into account the most recent parmbsc1 modifications^27,28^ introduced by the Barcelona Supercomputing Group to improve upon the parametrization of the backbone ε, ζ and glycosidic torsion angle χ dihedral angle force field parameters was adopted for the DNA. Umbrella sampling simulations were performed through the introduction of an external 1D harmonic bias potential, along a single reaction coordinate, defined as the distance between the centers-of-mass of the DNA molecules. The harmonic force constant for the umbrella sampling simulations was 5000 kJ mol^–1^ nm^–2^. The reaction coordinate was sampled in a range of 2 to 7 nm with a symmetric distribution of sampling windows, such that the window spacing was 0.1 nm.

**Table 1:** Studied Systems and Simulated Conditions^a^

System	*N*_bp_	*M*_w_ (g mol^–1^)	Number of water molecules, *N*_water_	Size of simulation box (nm)	Type & Number of cations	Number of Cl^–^ anions
L24Na	24	15600	107133	15.0	Na^+^: 295	203
L24Ca	24	15600	107279	15.0	Ca^2+^: 148	204
R65Na	65	42250	105105	15.0	Na^+^: 459	203
R65Ca	65	42250	105335	15.0	Ca^2+^: 230	204

a System acronyms in the first column denote topology (L- or R-), number of bps (24 or 65) and type of counterion used (Na^+^ or Ca^2+^).

For each sampling window, energy minimization of the system of DNA pairs, ions, and water solvent molecules was performed using a standard multistage protocol:^29−32^

a) Starting with the restrained minimization of the solute (DNA) while the solvent (water) and ions are unrestrained, several stages of 10000 steps of steepest descent minimization were performed with a maximum force tolerance termination criterion equal to 100.0 kJ mol^–1^ nm^–1^, each time gradually relaxing the position restraints imposed on DNA (e.g., from 25 to 0 kJ mol^–1^ nm^–2^), and b) continuing with the unrestrained optimization of the entire system (DNA and water and ions) which comprised two sequential stages of steepest descent and conjugate gradient.

Subsequently, each system was heated to 300 K and subjected to a dual-stage pre-equilibration procedure: with the first stage involving a short unrestrained MD simulation (∼100 ps) in the isothermal-isochoric *NVT* statistical ensemble, by making use of the Berendsen thermostat,^33^ and employing two separate heat baths for the solute and the solvent and ion systems in order to avoid the so-called “Hot Solvent/Cold Solute Problem”. In the second stage, the system was subjected to short unrestrained MD simulations (1 ns duration) in the isothermal–isobaric *NPT* ensemble by making use of the Nosé–Hoover thermostat,^34,35^ coupled with the Parrinello–Rahman barostat, with temperature *T* and pressure *P* fixed at their prescribed values of *T* = 300 K and *P* = 1 atm and time step equal to 1 fs.

Following equilibration, production simulations were performed for 50 ns with a 2 fs time step. All MD production simulations were conducted in the isothermal–isobaric (*NPT*) statistical ensemble by making use of the Nosé–Hoover thermostat coupled with the Parrinello–Rahman barostat^36^ to maintain temperature *T* and pressure *P* fixed at their prescribed values of *T* = 300 K and *P* = 1 atm. For the production simulations, a single heat bath was used for the entire system (solute and solvent, and ions). Normal temperature and pressure conditions with default temperature and pressure settings at 300 K and 1 atm, respectively, were used. The Particle Mesh Ewald (PME) method^37^ was used to treat electrostatics with a Fourier spacing of 0.12 nm. For van der Waals interactions, a cutoff distance equal to 1.2 nm was applied. The same distance (1.2 nm) was also used for the calculation of electrostatic Coulomb interactions in real space before switching to calculations in reciprocal (Fourier) space. All bonds were constrained by making use of the LINCS^38^ algorithm. The MD simulations were performed with the GROMACS software, version 2019.3.^39−41^ Following the completion of the MD production runs for all sampling windows, the weighted histogram analysis method (WHAM)^42,43^ was used for the reconstruction of the potential of mean force (PMF).

## 3 Results and Discussion

### i Effective Potentials

**Figure 1:**
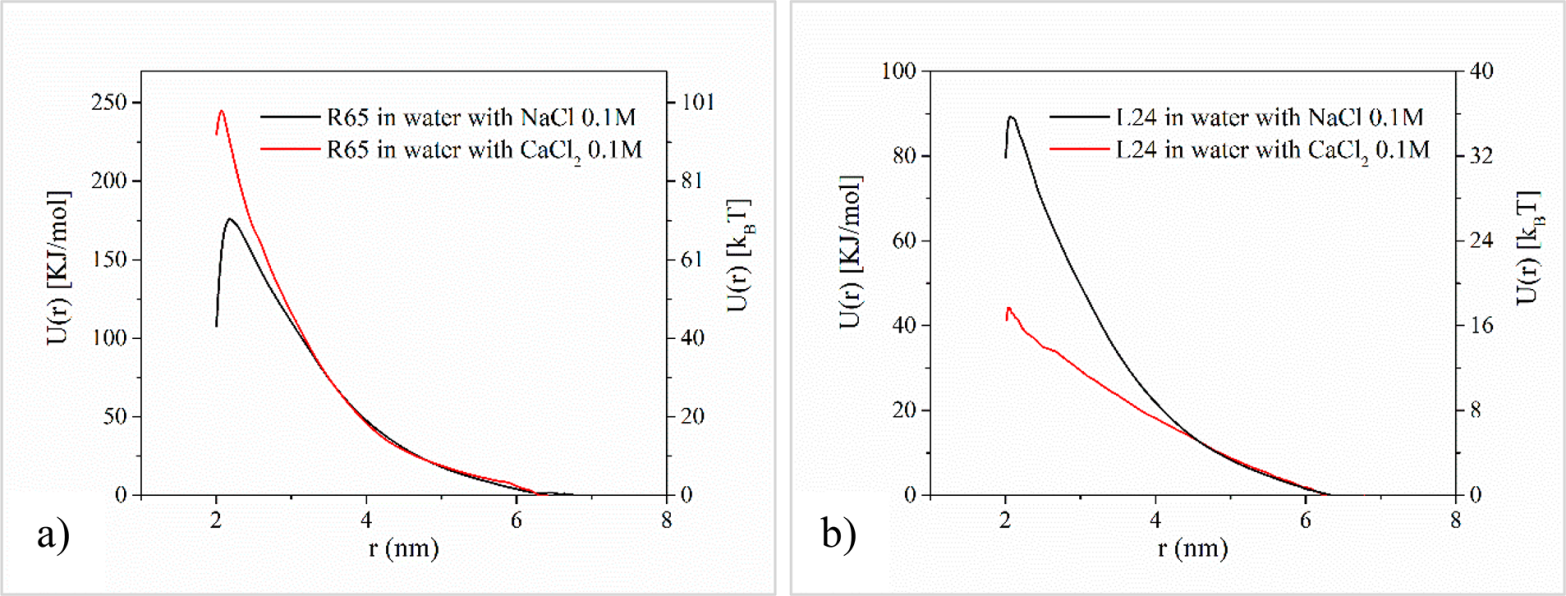
Effective isotropic potential between the centers of mass of a pair of a) 65 bp ds DNA minicircles and b) a pair of 24 bp ds DNA linear fragments.

**Figure 2:**
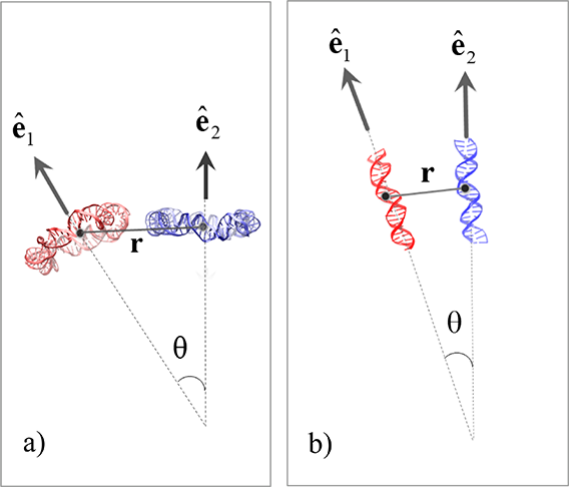
Schematic of the reaction coordinate **r** along which the effective interactions are estimated, as well as the relative orientation angle θ formed between the two eigenvectors corresponding to either the smallest or largest eigenvalue of the two respective gyration tensors of a pair of DNA molecules, with the former case applicable to DNA minicircle pairs and shown in panel a) and the latter case applicable to linear DNA fragment pairs and shown in panel b).

Effective pair interaction potentials have been computed on the basis of the reconstruction of the potential of mean force (PMF) from the umbrella sampling simulations by employing the weighted histogram analysis method (WHAM).^43^ A schematic showing the reaction coordinate along which the effective pair potentials are computed, defined as the center-of-mass separation of a pair of DNA molecules, is shown in Figure 2. The effective pair potential between the centers of mass of a pair of 65 bp ds DNA minicircles is shown in Figure 1a, and the respective umbrella histograms are shown in Figure S2 of the Supporting Information. Similarly, the effective pair potential between the centers of mass of a pair of 24 bp linear ds DNA is shown in Figure 1b, and the respective umbrella histograms are shown in Figure S2 of the Supporting Information. The effective pair potential, *V*_eff_(*r*), at a distance of *r* = 7.0 nm was chosen as a reference point and defined to zero in all cases. The effective interactions between the centers of mass of the DNA minicircles studied here can be reasonably expected to be decaying to zero at distances larger than 2·*R*_*g*,0_, where *R*_*g*,0_ ≃ 3.5 nm is the mean radius of gyration of the 65 bp minicircles in infinite dilution. In fact, previous, unbiased MD simulations of the DNA minicircle structures studied here indicate that the radial pair distribution function of the centers-of-mass of the two minicircles fluctuates around 1 at distances larger than 6.0 nm, yielding an effective potential estimated to be zero through a simple Boltzmann inversion calculation.^32^ In general, the overall qualitative shape of the computed effective pair potentials of Figure 1a exhibits features similar to the respective potentials of semiflexible unknotted, nonconcatenated rings modeled by using the well-known bead–spring model of Kremer and Grest.^45,46^ A strong repulsion persists between DNA minicircles for the conditions studied here, and the magnitude of the effective potential in the sodium chloride solution is on the order of 100 kJ/mol, or approximately 15 kJ/mol per helical turn at the closest center-of-mass distance investigated/sampled with the umbrella sampling simulations, equal to 2.0 nm. A much stronger repulsion is exerted between DNA minicircles in the calcium chloride solution at the same reference center-of-mass distance of 2 nm. A single, pronounced principal peak appears in both cases located at a distance close to 2.2 nm for the case of sodium counterions. A much narrower peak develops for the case of the divalent counterion solution, appearing at slightly shorter center-of-mass distances, close to 2.1 nm.

An interesting comparison can be drawn between the different DNA topologies studied here, especially with regard to the contrasting effects of the divalent counterions on the effective pair potentials. It appears that in the presence of divalent calcium ions, the effective pair potential between linear DNA chains is consistently lower than the respective one for monovalent sodium ions for center-of-mass distances up to approximately 4.5 nm. For larger distances, however, the two effective potential curves approach one another closely, becoming practically indistinguishable. A markedly different effect can be observed in the case of DNA minicircles though: at short separations, the repulsion exerted in the presence of calcium ions between a DNA pair is significantly stronger compared to the respective one in the presence of sodium ions, despite the more effective screening effected by divalent ions. This counterintuitive effect can be observed up to center-of-mass separations of about 3.0 nm, while thereafter it subsides and the effective potential curves corresponding to the two counterions become practically indistinguishable. An explanation of this seemingly surprising effect lies in the close observation of the favorable relative orientations that are adopted by DNA minicircles as a function of their center-of-mass separation, as well as in the associated variations in the ionic atmosphere, and is exposed in more detail in the next two sections.

### ii Pairwise Intermolecular Orientation Correlations

**Figure 3:**
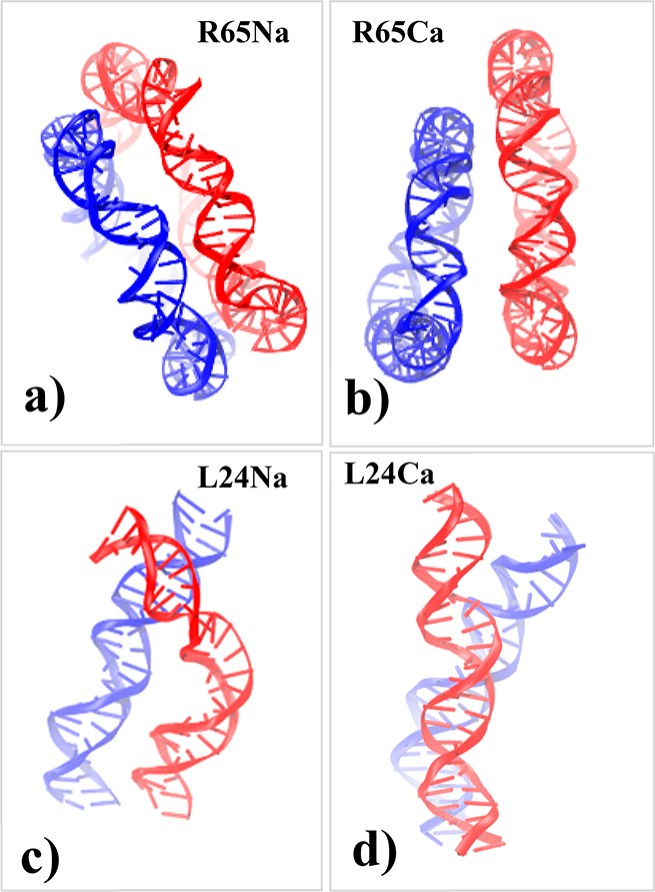
Typical atomistic snapshots of the instantaneous configuration of pairs of DNA molecules restrained at a center-of-mass distance of 2.0 nm: a) case of 65 bp DNA minicircles in a 0.1 M NaCl solution (system R65Na in Table 1), b) case of 65 bp DNA minicircles in a 0.1 M CaCl_2_ solution (system R65Ca in Table 1), c) case of 24 bp linear DNA fragments in a 0.1 M NaCl (system L24Na in Table 1), and d) case of 24 bp linear DNA fragments in a 0.1 M CaCl_2_ solution (system L24Ca in Table 1). The respective instantaneous orientation angles are 5.7°, 21.3°, 23.5°, and 27.8°. The VMD software was used for visualization.

Important insight into the counterintuitive effects of the counterion valency on the effective potentials can be gained by considering the typical conformations adopted by pairs of DNA minicircles and linear DNA chains as their center-of-mass separation distance increases. Indicative snapshots of the relative configurations of pairs of DNA minicircles (systems R65Na and R65Ca in Table 1), as well as linear DNA fragments (systems L24Na and L24Ca in Table 1) along varying center-of-mass distances are depicted in Figure 3, as well as in Figures S3–S5 of the Supporting Information. Furthermore, the relative angular orientation of the DNA molecule pairs is quantified in terms of the angle θ formed between the two eigenvectors **p**_1_, with **p**_2_ corresponding to the largest eigenvalue of the two respective gyration tensors as follows:
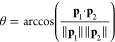
1The radius of gyration tensor for each DNA molecule is defined by the dyadic  with **r**_*i*_ denoting the position vector of atom *i* in a frame of reference with origin set at the center-of-mass of the molecule. Upon transformation to a principal axis system that diagonalizes **S** such that , where the set {λ_1_^2^, λ_2_^2^, λ_3_^2^} denotes the eigenvalues (principal moments) of the tensor arranged in descending order, i.e., λ_1_^2^ ≥ λ_2_^2^ ≥ λ_3_^2^.

**Figure 4:**
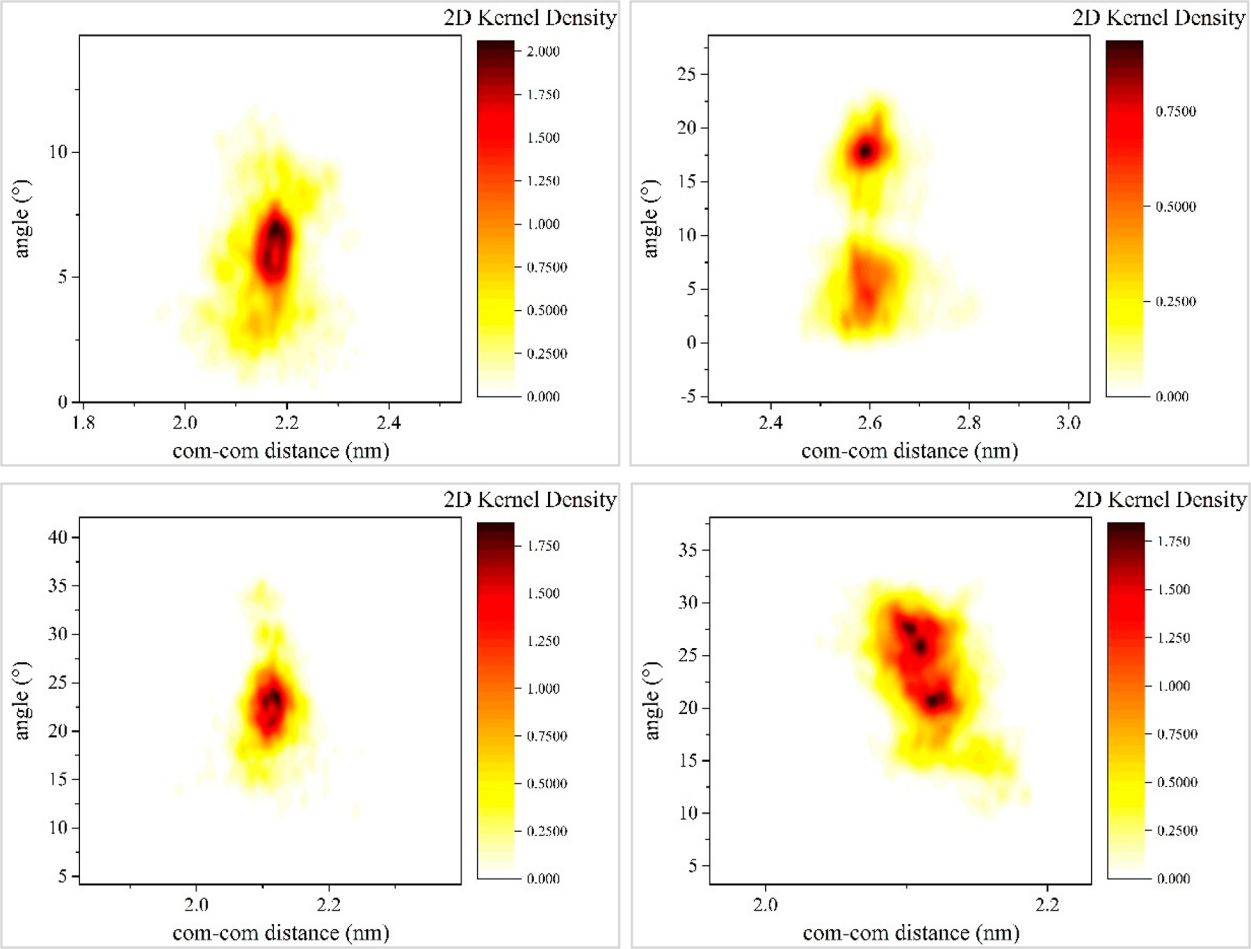
Probability density function of the average orientation angles between the eigenvectors of a pair of DNA molecules restrained at small center-of-mass distances (i.e., 2.0–2.6 nm): a) case of 65 bp DNA minicircles in a 0.1 M NaCl solution (system R65Na in Table 1), b) case of 65 bp DNA minicircles in a 0.1 M CaCl_2_ solution (system R65Ca in Table 1), c) case of 24 bp linear DNA fragments in a 0.1 M NaCl (system L24Na in Table 1), b) case of 24 bp linear DNA fragments in a 0.1 M CaCl_2_ solution (system L24Ca in Table 1). Eigenvectors corresponding to either the largest or smallest eigenvalue of the respective gyration tensors are employed for the calculation in the case of linear DNA or minicircle DNA, respectively.

The dependence of the estimated relative angular orientation angles on the center-of-mass distances is illustrated in Figure 4, as well as in Figures S6–S8 of the Supporting Information, for the cases of DNA minicircles and linear chains. Results are shown in the form of probability density functions calculated from the respective scatter points. To this end, a 2D kernel density estimation (KDE) with a Gaussian kernel probability density function^47^ was employed in order to determine density values, as follows:  with **x** and **x**′ denoting the corresponding vectors of values of a correlated pair of observables, namely, the instantaneous average orientation angle and center-of-mass separation. The resulting population densities are visualized with different colors by use of a color map, as shown in Figure 4. Upon a close inspection of the correlations between the instantaneous relative orientation angle and center-of-mass separation for the case of linear DNA fragments, it becomes clear that in small center-of-mass separations the two linear DNA chains deviate from their initial parallel-placement by rotating relative to one another, so as to adopt orientations that result in minimizing the proportion of their surfaces located in close proximity with respect to one-another. Such relative orientations minimize the intermolecular electrostatic and steric repulsion. Interestingly enough, in small center-of-mass separations, linear DNA fragments can also exhibit significant backbone bending motions, as shown in Figure 3. Larger center-of-mass separations offer more leeway for relative rotation of the DNA chains pair, as reflected by the increase in the range of relative orientation angles in Figures S6–S8. Rotations resulting in an instantaneous end-to-end approach of the two linear chains can be observed in sufficiently large center-of-mass separations (i.e., greater than 3.0 nm). In calcium chloride solutions, the more efficient screening of backbone phosphate charges allows for noticeably larger rotations of the linear chains relative to one another over the entire range of center-of-mass separations sampled, as clearly shown by the relative angle probability density plots. For the case of DNA minicircle pairs, evidently smaller relative orientation angles, as compared to linear chains, can be observed over the entire range of center-of-mass separation distances sampled. In the presence of sodium counterions, minicircles maintain an almost parallel relative orientation for center-of-mass separations smaller than their mean radius of gyration at infinite dilutions but exhibit an increased rotational motion as their distance increases further. In a similar fashion to their linear counterparts, due to the more efficient charge screening imparted in the presence of calcium counterions, DNA minicircles exhibit increased rotational motion in the entire range of center-of-mass separations studied here. Indicative snapshots of the instantaneous relative orientations adopted by the pairs of DNA minicircles in the divalent ion solution show not only increased rotational motion but also out-of-plane bending motion arising at separations approaching the mean radius of gyration. As a result, an increased degree of structural deformation of DNA minicircles, as compared to their starting configurations, is observed in the presence of divalent cations.

**Figure 5:**
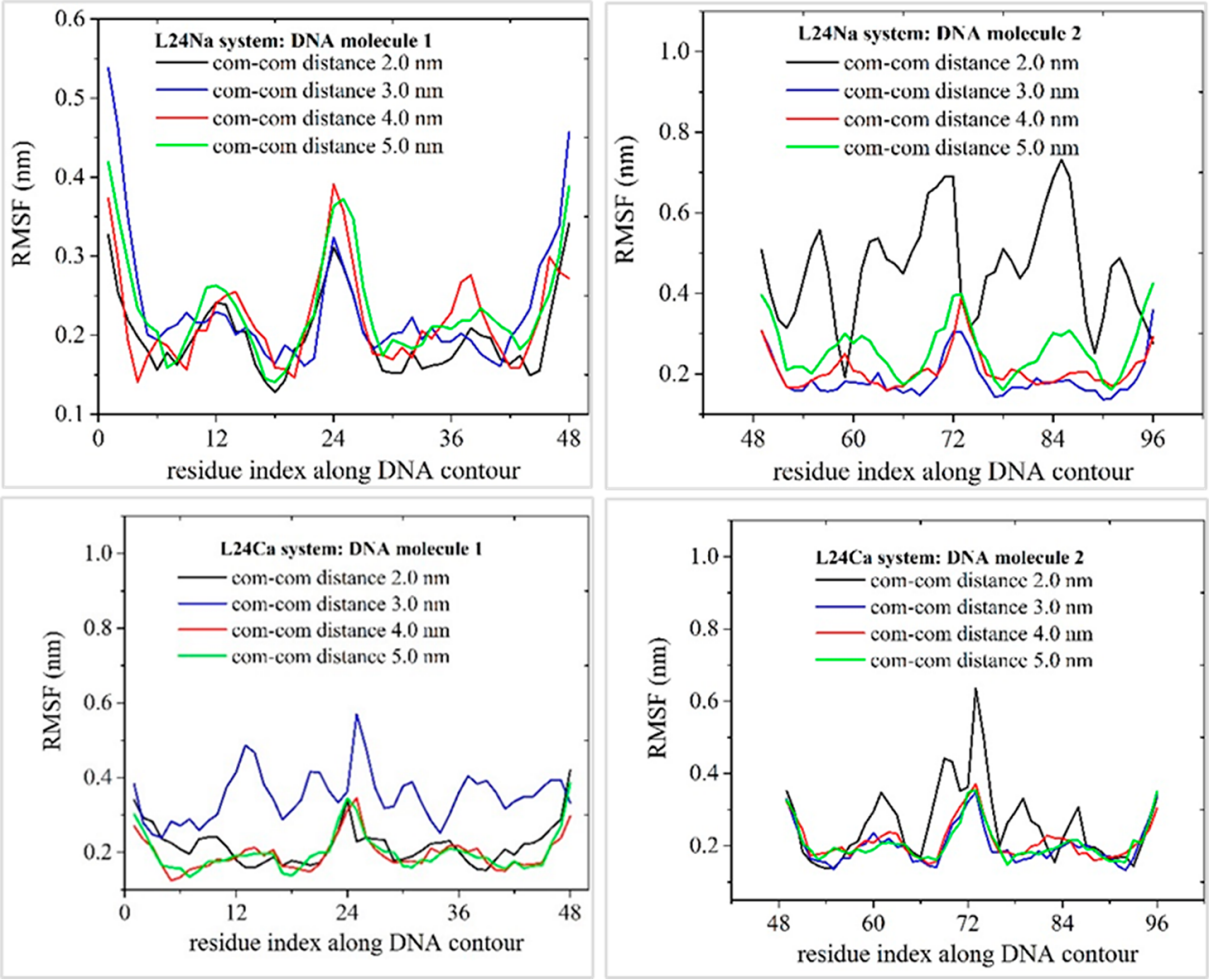
Time-averaged RMSF calculated for the C3′ backbone atoms along the DNA contour, as a function of the residue (base pair) index along the DNA strand. Residue indices for both DNA strands are shown, following the 5′-3′ and 3′-5′ strand convention. Results are shown for the pair of 24 bp ds DNA linear fragments (systems L24Na and L24Ca in Table 1).

**Figure 6:**
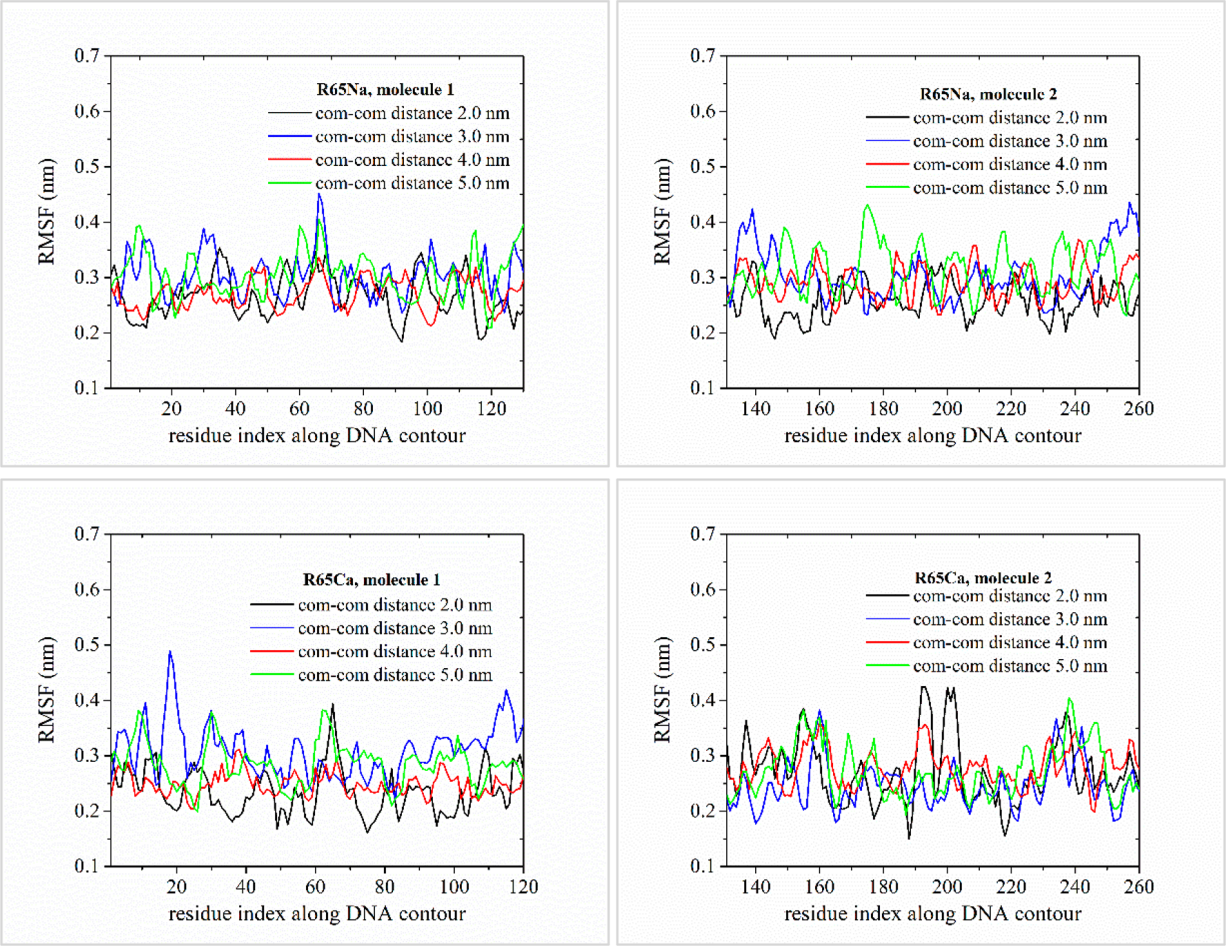
Time-averaged RMSF calculated for the C3′ backbone atoms along the DNA contour, as a function of the residue (base pair) index along the DNA strand. Residue indices for both DNA strands are shown, following the 5′-3′ and 3′-5′ strand convention. Results are shown for the pair of 65 bp ds DNA minicircles (systems R65Na and R65Ca in Table 1).

A more detailed characterization of the extent of structural deformation of the DNA backbone is obtained by the computation of the root-mean-square fluctuation (RMSF), a commonly employed bioinformatics metric that provides an estimate of the positional fluctuations of an atom group within a certain period of time. The RMSF is computed after the optimal superposition or alignment of the atomic coordinates of each instantaneous conformation to a reference structure (the starting configuration is chosen here)^48−50^ and typical results are shown in Figures 5 and 6.

Pronounced structural fluctuations in the free end region of linear DNA fragments are evident in small center-of-mass separations for the case of sodium counterions. As the center-of-mass separation of linear DNA fragments increases further, free end structural fluctuations are significantly suppressed in NaCl solution. On average, the backbone structural fluctuations of linear DNA along its contour are slightly increased with increasing center-of-mass separation in the range 3.0–5.0 nm. Notably, in the presence of calcium counterions, free end fluctuations are markedly diminished. In the case of DNA minicircles, similar order of magnitude values of the RMSF can be observed as compared to their linear counterparts.

### iii Ionic Atmosphere

**Figure 7:**
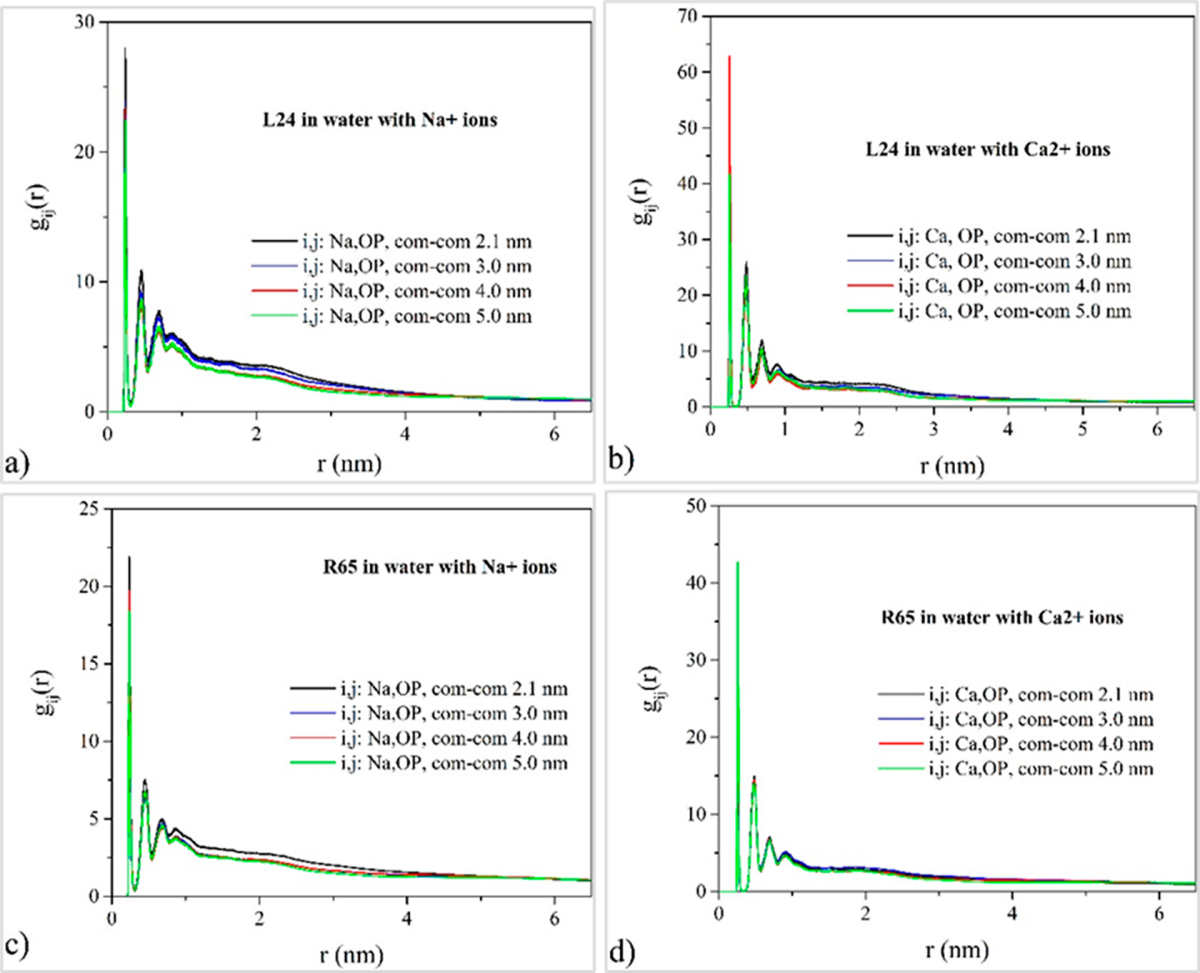
MD simulation results for the radial pair distribution function between salt counterions and phosphate group oxygens (results have been averaged for the O1P and O2P type atoms following the amber force field naming convention) and their dependence on DNA topology and counterion valency. Top row corresponds to the system of two linear DNA molecules with a) Na^+^ counterions and b) Ca^2+^ counterions. Bottom row corresponds to the system of two DNA minicircles with c) Na^+^ counterions and d) Ca^2+^ counterions.

**Figure 8:**
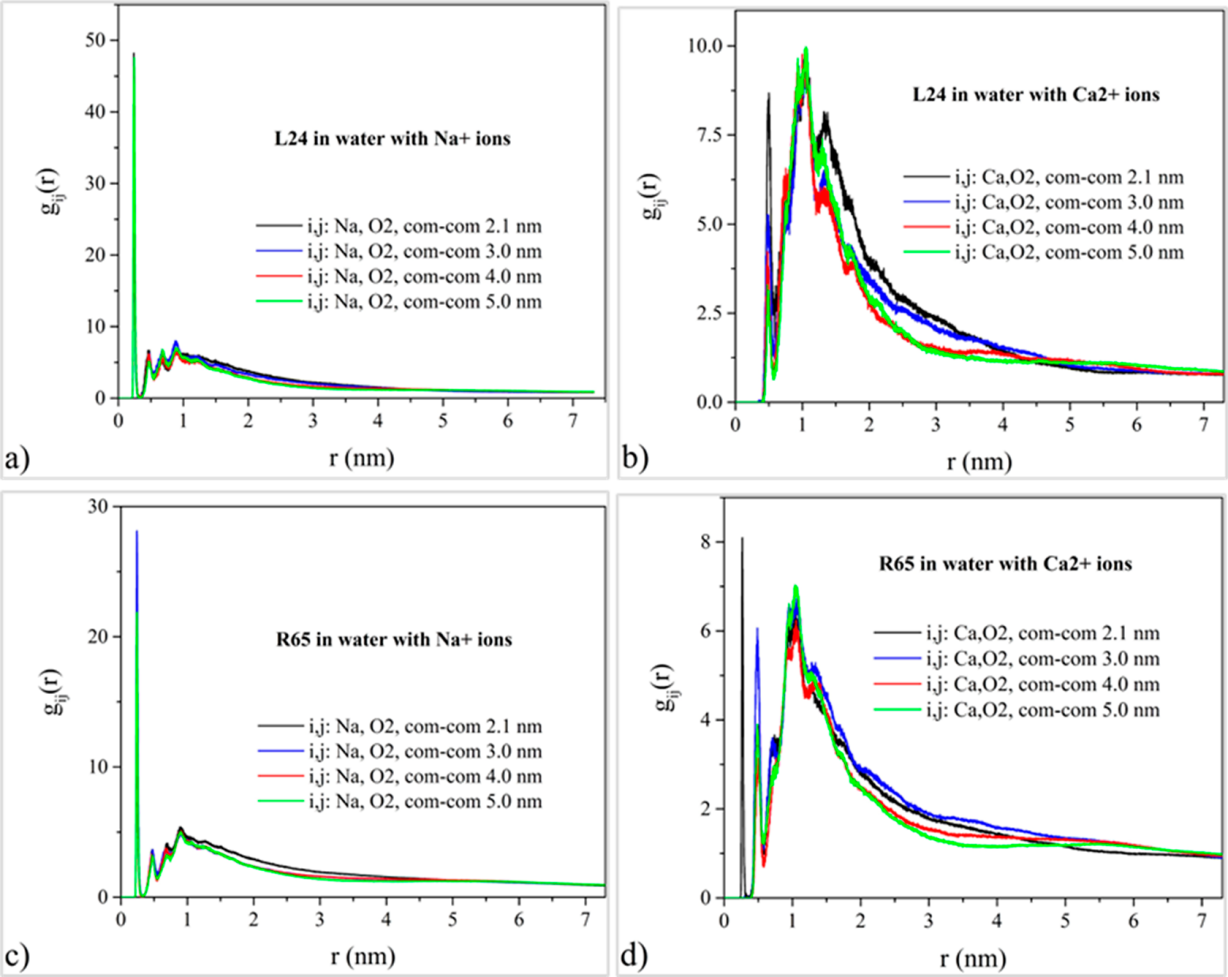
MD simulation results for the radial pair distribution function between salt counterions and minor groove O2 oxygens and its dependence on DNA topology and counterion valency. Top row corresponds to the system of two linear DNA molecules with a) Na^+^ counterions and b) Ca^2+^ counterions. Bottom row corresponds to the system of two DNA minicircles with c) Na^+^ counterions and d) Ca^2+^ counterions.

**Figure 9:**
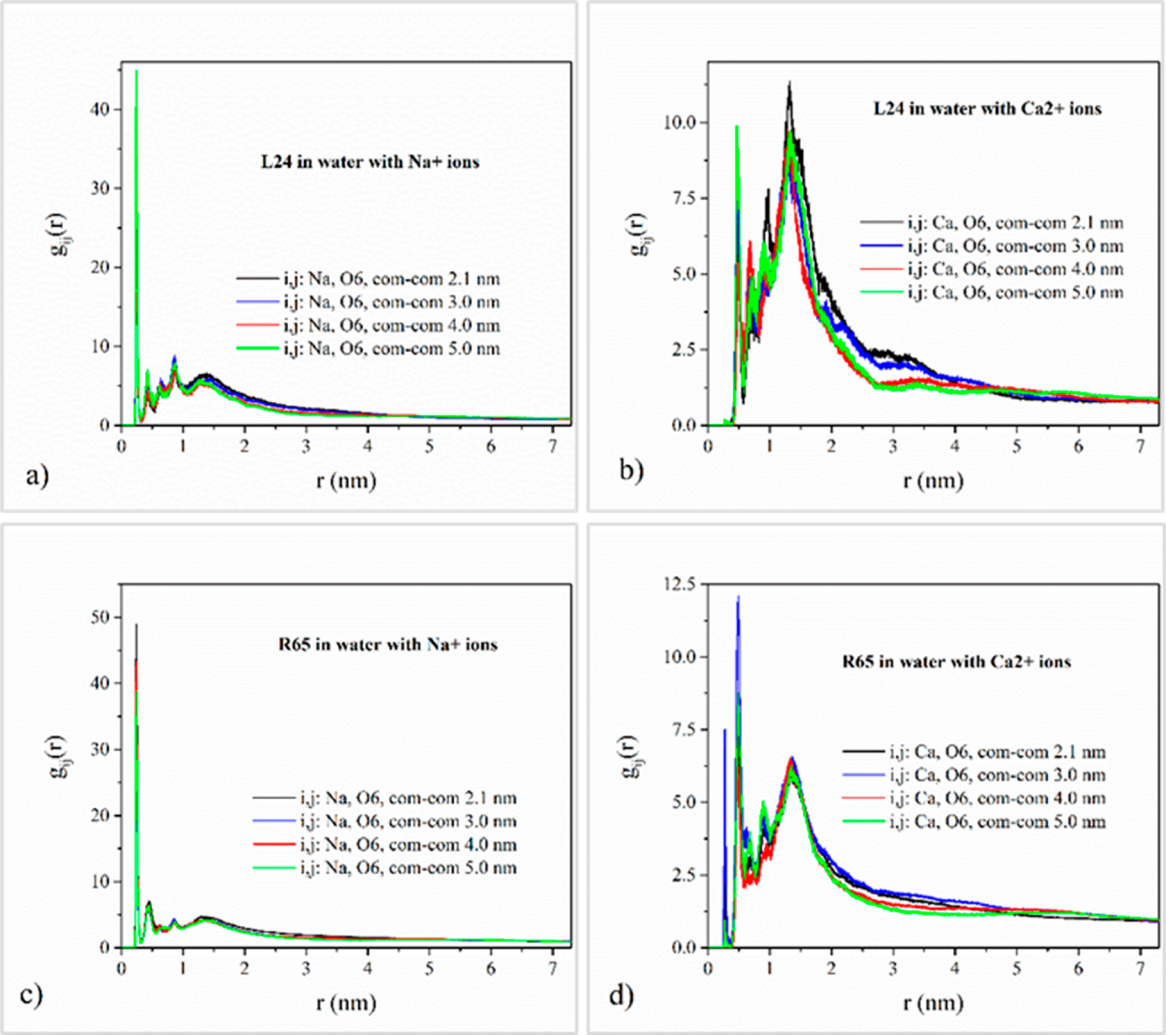
MD simulation results for the radial pair distribution function between salt counterions and major groove O6 oxygens and its dependence on DNA topology and counterion valency. Top row corresponds to the system of two linear DNA molecules with a) Na^+^ counterions and b) Ca^2+^ counterions. Bottom row corresponds to the system of two DNA minicircles with c) Na^+^ counterions and d) Ca^2+^ counterions.

In this section, the ionic atmosphere surrounding DNA is investigated by computing the radial pair distribution function *g*_*ij*_(*r*) of sodium and calcium counterions relative to selected DNA backbone and groove atoms in order to determine preferential binding sites and facilitate the understanding of the effects of counterion correlations on the effective pair potentials for DNA. To this end, the radial pair distribution functions of the sodium and calcium counterions relative to the phosphate group oxygen atoms, jointly denoted here as OP atoms, are computed, and typical results are presented in Figure 7. For the quantification of the ionic atmosphere in the DNA grooves, MD simulation results for the radial pair distribution function between salt counterions and major and minor groove oxygen atoms are depicted in Figures 8 and 9. For the case of sodium cations, strong ionic condensation in the vicinity of the OP atoms is evident by the presence of intense peaks at a distance of approximately 0.24 nm. This closest contact distance between the DNA strand backbone and sodium cations is in close agreement with those in previous experimental and modeling studies.^66,67^ Secondary peaks occurring at larger distances indicate solvent-mediated sodium–phosphate contacts. This type of condensation in the vicinity of the DNA strand backbone becomes weaker as the center-of-mass separation between DNA pairs increases for both linear and minicircle DNA. A much stronger condensation occurs for the case of calcium ions in the neighborhood of the DNA strands, as indicated by the markedly higher intensity of the principal peak observed at approximately 0.25 nm. Solvent mediated calcium–DNA contacts are reflected in secondary peaks. A moderately weaker condensation effect is evident for DNA minicircles compared to their linear counterparts, regardless of the ionic valency, and the effect is more pronounced for divalent calcium ions.

In the vicinity of the minor groove, sodium counterions exhibit a strong condensation effect for both DNA topologies studied, contrary to the case of calcium counterions, as depicted in Figure 8. The counterion condensation in the minor groove is consistently stronger for linear chains regardless of the ionic valency. Broad, diffuse peaks are evident for the case of calcium, reflecting the presence of calcium ions hydrated by multiple water molecules, as indicated by previous studies. A strong counterion release effect is obvious for the case of calcium, as the center-of-mass separation of the DNA pair increases, and this effect becomes more pronounced for linear chains than minicircles. Ionic condensation in the major groove is also substantial for the case of sodium ion, as indicated by the plots of the radial pair distribution function of the counterions relative to the major groove oxygen O6, shown in Figure 9. Moderately weaker major groove condensation of sodium occurs for the case of minicircles compared to linear chains. Calcium condensation in the major groove is stronger than the respective condensation in the minor groove but still less significant than the respective condensation close to the DNA strands.

**Figure 10:**
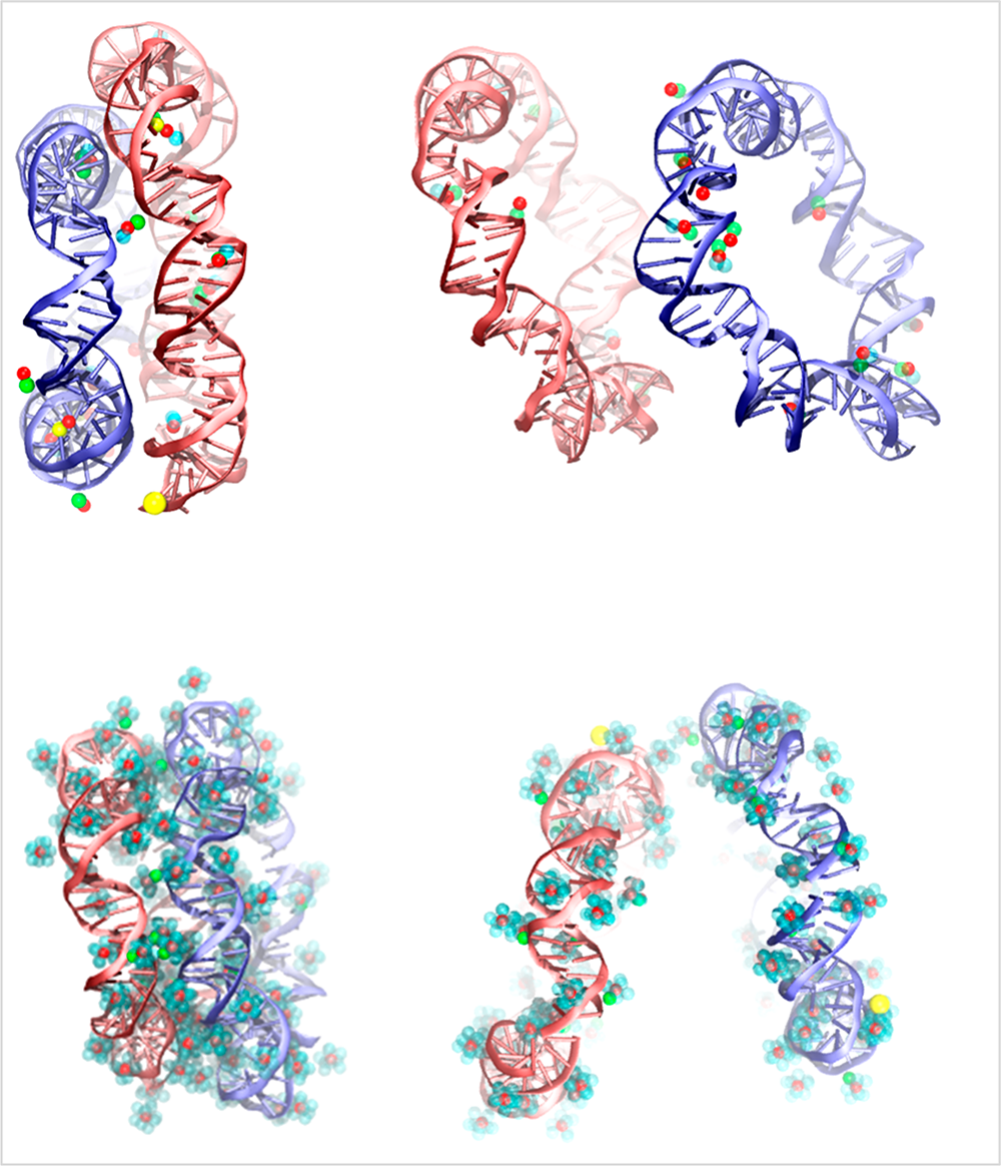
Typical atomistic snapshots of the instantaneous configurations of a pair of 65 bp DNA minicircles and their associated ionic atmosphere: top row figures correspond to the NaCl 0.1 M solution, depicting short (2.0 nm) and large (5.0 nm) center-of-mass distances, while bottom row figures correspond to the CaCl_2_ 0.1 M solution, depicting short (2.0 nm) and large (5.0 nm) center-of-mass distances. Graphical representation of the ionic atmosphere is as follows: sodium and calcium cations are shown in bright red color, chloride anions are shown in yellow color, and water oxygen atoms associated with the calcium or sodium cations within the closest contact radius of the respective cation with DNA strands or grooves are shown in light blue color.

A detailed observation of the ionic atmosphere associated with DNA minicircles offers an interesting explanation of the unexpected effects of increased ionic valency on the effective potentials, shown in Figure 1. To this end, snapshots of the instantaneous configurations of a pair of 65 bp DNA minicircles and their associated ionic atmosphere are shown in Figure 10, for increasing center-of-mass separations. In addition to the ions, water oxygen atoms that are associated with the condensed calcium or sodium cations within a distance equal to the closest contact radius of the respective cation with DNA strands or grooves are also shown. Apparently, a much larger population of water molecules is located within the first contact shells of calcium ions with DNA strands. Respective results of the radial pair distribution function of sodium and calcium counterions computed relative to water oxygen atoms are provided in Figure S10 of the Supporting Information. In order to elaborate further on the hydration structure of different counterions, the coordination numbers with respect to water oxygen atoms in the first hydration shell as well as the hydration shell radius are reported in Table S1. From the form of the first hydration shell peak, it becomes clear that calcium ions are more tightly bound to their surrounding water molecules, as compared to sodium ions, and are also surrounded by a larger population of water molecules. Due to the presence of these hydrated calcium ion clusters and the formation of an interconnected network of hydrogen bonds between them, the relative orientations that can be adopted by the DNA minicircles become significantly restricted, resulting in a notable loss of their conformational entropy. Additional entropy losses occur for the counterions and solvent, which are mostly orientational and result from the short-range order induced in the water molecules surrounding the counterion and the reduction in the volume of the configuration space available for the formation of hydrogen bonds with neighboring bulk water molecules.

Despite the fact that similar solvation structure and counterion induced entropy losses also persist for the case of linear DNA pairs in the presence of calcium, a larger configuration space is available to linear DNA fragments because of their distinctive ability to rotate relative to one another and exhibit more pronounced bending due to the presence of free ends, thereby adopting relative orientations that minimize electrostatic and steric repulsions. In addition, due to the more effective charge screening induced by the divalent counterions, the range of rotational motion of linear DNA increases further compared to that of the monovalent counterion case.

Therefore, in the case of the linear DNA fragments, the entropic gain related to the increased range of rotational and free end bending motion, compounded by the more effective charge screening of the divalent ion, compensates for the condensation-related and calcium counterion-hydration-induced entropic losses. As a result, the short-range effective potential exerted between linear DNA molecules in the presence of calcium is substantially weaker than the respective one in the presence of sodium.

On a further note, the existence of a maximum in the effective potentials depicted in Figure 1 correlates with the entropic gain effected by the process of counterion release, which occurs for increasing center-of-mass separations. In the opposite direction, a strong condensation of the counterions on the DNA occurs as the center-of-mass separation becomes very small, so as to reduce the electrostatic repulsion between DNA molecules and results in the decrease of the effective interactions as the center of mass separation diminishes. A related phenomenon has been in fact demonstrated for the effective interactions of charged polyelectrolytes, which, however, are dominated by the entropic contributions of the trapped counterions.^51^

For the case of the divalent calcium counterion, a much stronger counterion release effect occurs, as compared to the sodium counterion, and this is clearly evident both in the vicinity of the DNA strands (Figure 7) as well as in the major and minor groove regions (Figures 8 and 9 and Figure S9). As a result, the respective maximum peak in the effective potential in the calcium chloride solution is less wide than the respective one in the sodium chloride.

Lastly, it is worth noting that there has been a lot of research effort directed to the improvement of ionic force fields in the regime of finite ionic concentrations (>0.1 M). Notable improvements have been accomplished either by employing multiobjective optimization techniques, e.g., simultaneous optimization of single-ion and ion-pair properties,^52−54^ or by introducing a concentration dependent dielectric permittivity and deriving effective ionic potentials that capture many-body effects.^55^ For the case of calcium ions, indicative simulations have been performed by use of a modified ionic field^53^ and sample conformations of DNA minicircles are shown in Figure S11. The conformations of DNA minicircles and their relative orientations remain qualitatively similar to those reported by use of the standard amber ff, and no notable change is observed. Further, we also report the respective radial pair distributions of calcium ions relative to the DNA phosphate oxygens as well as minor and major groove oxygens in Figure S12. Despite the presence of stronger major peaks in Figure S11 as compared to Figures 7–9, this effect is substantially subdued when the number of ions contained within the first shell is estimated, as obtained by integration of the *g*(*r*) over the first peak. For higher ionic concentrations, the use of such improved force fields is expected to be indispensable, but for the low ionic concentrations studied here, no major effects are induced.

## 4 Conclusions

The effects of molecular topology, namely, linear and circular, as well as counterion valency, on the ensuing pairwise effective interactions between moderately short, stiff DNA molecules in an unlinked state have been computationally investigated by means of Umbrella Sampling simulations. Purely repulsive effective interactions persist between the linear and minicircle DNA molecules studied here, for both ionic solutions investigated (i.e., NaCl and CaCl_2,_ of concentration 0.1 M). A counterintuitive effect of the divalent calcium ions on the effective interactions between DNA minicircles is observed, as the short-range effective repulsion in the 0.1 M CaCl_2_ solution increases significantly compared to the respective 0.1 M NaCl solution, despite the more effective charge screening of the divalent counterion. On the contrary, in the case of linear DNA, the opposite effect occurs and can be attributed to the propensity of linear DNA fragments to rotate relative to one another and to exhibit more pronounced bending due to presence of free ends, so as to minimize electrostatic and steric repulsions.

Distinct binding modes are evident in the condensation of each counterion type studied, with the monovalent sodium ions exhibiting a strong preferential binding in the vicinity of the DNA grooves, while the calcium ions are less frequently found within the groove region and are mostly located closer to the DNA strands. Sodium counterion binding in the DNA grooves occurs mostly in the minor groove for the case of the linear DNA, which could be attributed to its AT-rich base sequence and the presence of strongly electronegative AT pockets, while for minicircles, major groove binding is preferred. In fact, experimental studies point out that the alkali metal ions, including Na and K, are mostly located in the minor groove of AT-rich sequences, whereas divalent cations preferentially bind in the major groove of CG-rich base sequences.^56−59^ In agreement with experimental observations, theoretical studies suggest that the electrostatic potential of the minor groove of an AT base pair step is most negative, very closely followed by the GC major groove, the GC minor groove and, finally, the AT major groove.^60^ Thus, the strong binding preference of sodium for the minor groove of the linear DNA and the major groove of the DNA minicircles that is observed herein could possibly be related to this base-specific electronegativity ordering suggested by theoretical models. In a similar vein, some previous single molecule MD studies have pointed out increased occupancy of Na ions within the minor groove regions that are localized at AT base pair steps.^61−63^

In this study, we focused on the computational investigation of the effective interactions between moderately short linear and circular ds DNA in an unlinked state and within a pure aqueous solution environment. Nonetheless, mixed solvents, comprising, for example, binary aqueous mixtures with ethanol and polyethylene glycol, have recently attracted increased research interest, with recent experimental studies exemplifying the possibility of solvent induced conformational transitions, occurring not only on a local base-pair level but also affecting the overall size of DNA structures as complex as the DNA kinetoplast. Consequently, varying the solvent quality and examining the effect of catenation constitute another area of great practical and theoretical importance where our future work is directed to.^64,65^

## Supporting Information Available

Typical atomistic snapshots of the initial configuration of pairs of 24 bp linear DNA fragments and 65 bp DNA minicircles, umbrella histograms for biased simulations of pairs of 24 bp linear DNA fragments and 65 bp DNA minicircles, probability density function of the average orientation angles between the eigenvectors, MD simulation results, typical atomistic snapshots of the instantaneous configurations of a pair of 24 bp linear DNA fragments ; tables of estimated radii and RMSF estimates from MD simulations (PDF)
