# Supplementary material for: Effective Interactions between Double-Stranded DNA Molecules in Aqueous Electrolyte Solutions: Effects of Molecular Architecture and Counterion Valency
Source: J Phys Chem B. 2023 Jul 26;127(31):6969–81. doi: 10.1021/acs.jpcb.3c02216 (PMC10424236; doi:10.1021/acs.jpcb.3c02216)
Supplement: Supplementary file 1 — jp3c02216_si_001.pdf [file jp3c02216_si_001.pdf]

Supporting Information for:

“Effective interactions between double-stranded DNA molecules in aqueous electrolyte solutions: effects of molecular architecture and counterion valency”

Terpsichori S. Alexiou, Christos N. Likos

Faculty of Physics, University of Vienna, Boltzmanngasse 5, 1090 Vienna, Austria

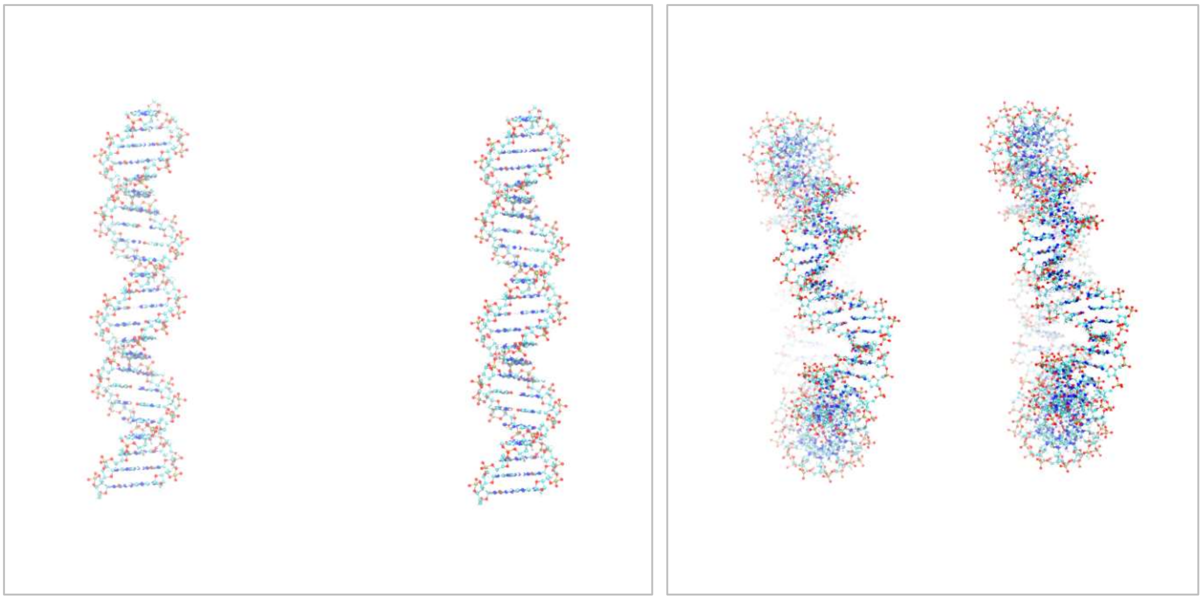

Figure S1. Typical atomistic snapshot of the initial configuration of the initial configuration of pairs of 24bp linear DNA fragments and 65bp DNA minicircles. The VMD software was used for visualization.

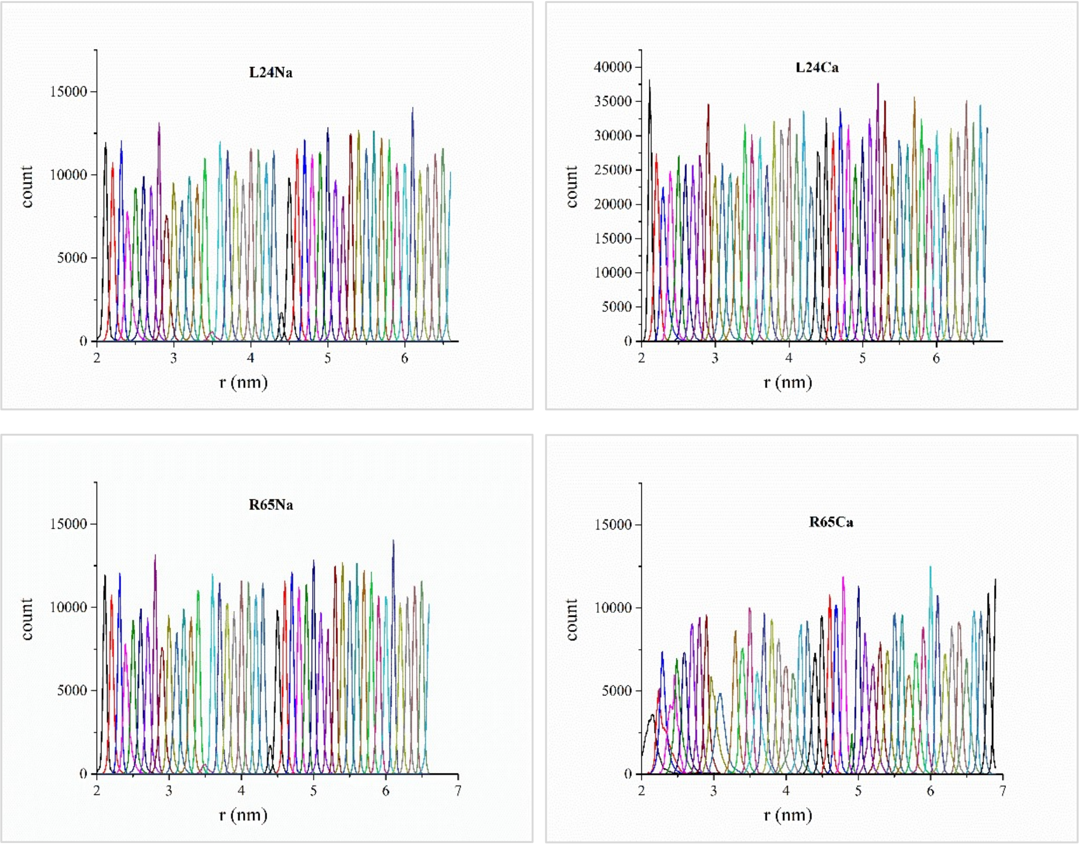

Figure S2. Umbrella histograms for biased simulations of pairs of 24bp linear DNA fragments and 65bp DNA minicircles.

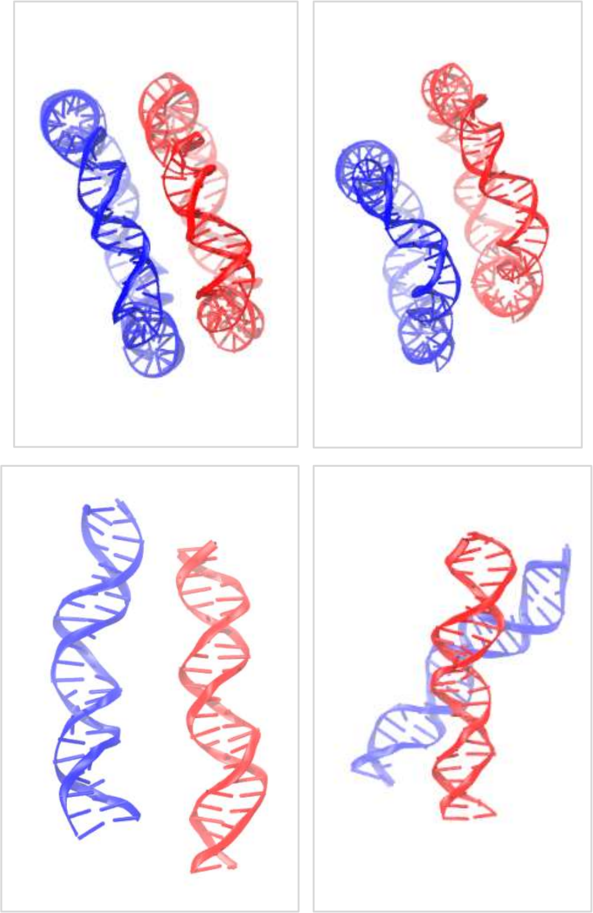

Figure S3. Typical atomistic snapshots of the instantaneous configuration of pairs of DNA molecules restrained at a center-of-mass distance of 3.0 nm: a) case of 65bp DNA minicircles in a 0.1M NaCl solution (system R65Na in Table 1), b) case of 65bp DNA minicircles in a 0.1M $CaCl_2$ solution (system R65Ca in Table 1), c) case of 24bp linear DNA fragments in a 0.1M NaCl (system L24Na in Table 1), d) case of 24bp linear DNA fragments in a 0.1M $CaCl_2$ solution (system L24Ca in Table 1). The respective instantaneous orientation angles are 15.7°, 16.5°, 17.0°, and 43.7° . The VMD software was used for visualization.

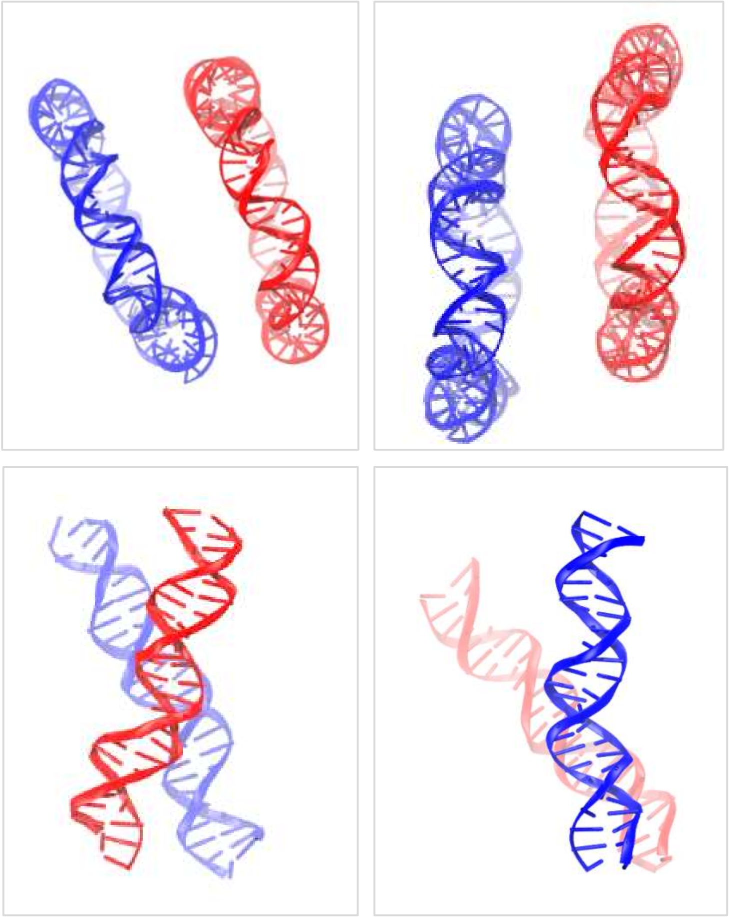

Figure S4. Typical atomistic snapshots of the instantaneous configuration of pairs of DNA molecules restrained at a center-of-mass distance of 4.0 nm: a) case of 65bp DNA minicircles in a 0.1M NaCl solution (system R65Na in Table 1), b) case of 65bp DNA minicircles in a 0.1M $CaCl_2$ solution (system R65Ca in Table 1), c) case of 24bp linear DNA fragments in a 0.1M NaCl (system L24Na in Table 1), d) case of 24bp linear DNA fragments in a 0.1M $CaCl_2$ solution (system L24Ca in Table 1). The respective instantaneous orientation angles are 15.7°, 16.5°, 17.0°, and 43.7° . The VMD software was used for visualization.

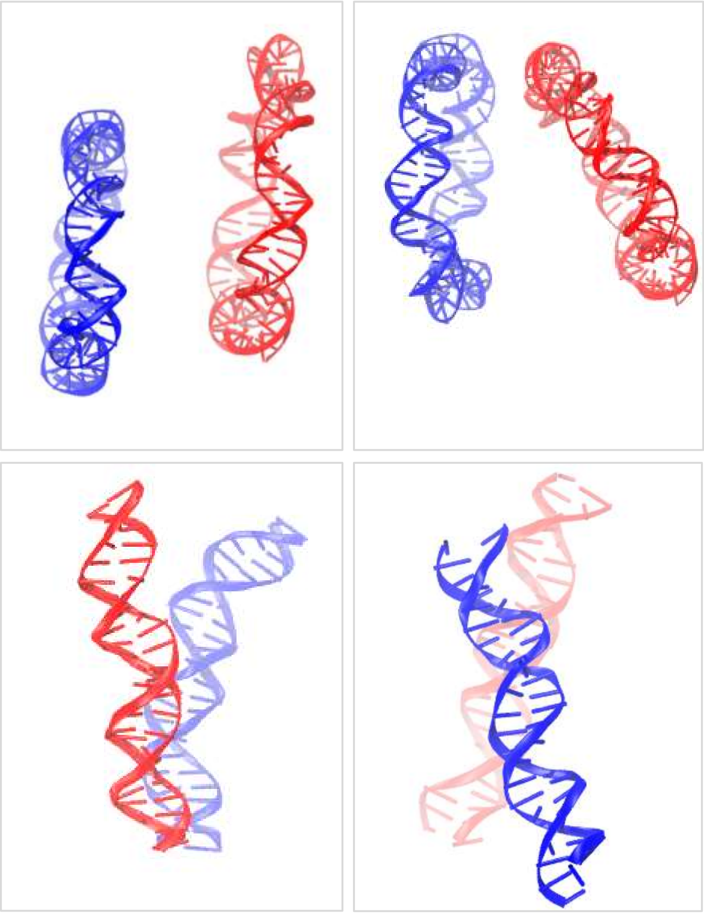

Figure S5. Typical atomistic snapshots of the instantaneous configuration of pairs of DNA molecules restrained at a center-of-mass distance of 5.0 nm: a) case of 65bp DNA minicircles in a 0.1M NaCl solution (system R65Na in Table 1), b) case of 65bp DNA minicircles in a 0.1M $CaCl_2$ solution (system R65Ca in Table 1), c) case of 24bp linear DNA fragments in a 0.1M NaCl (system L24Na in Table 1), d) case of 24bp linear DNA fragments in a 0.1M $CaCl_2$ solution (system L24Ca in Table 1). The respective instantaneous orientation angles are 15.7°, 16.5°, 17.0°, and 43.7° . The VMD software was used for visualization.

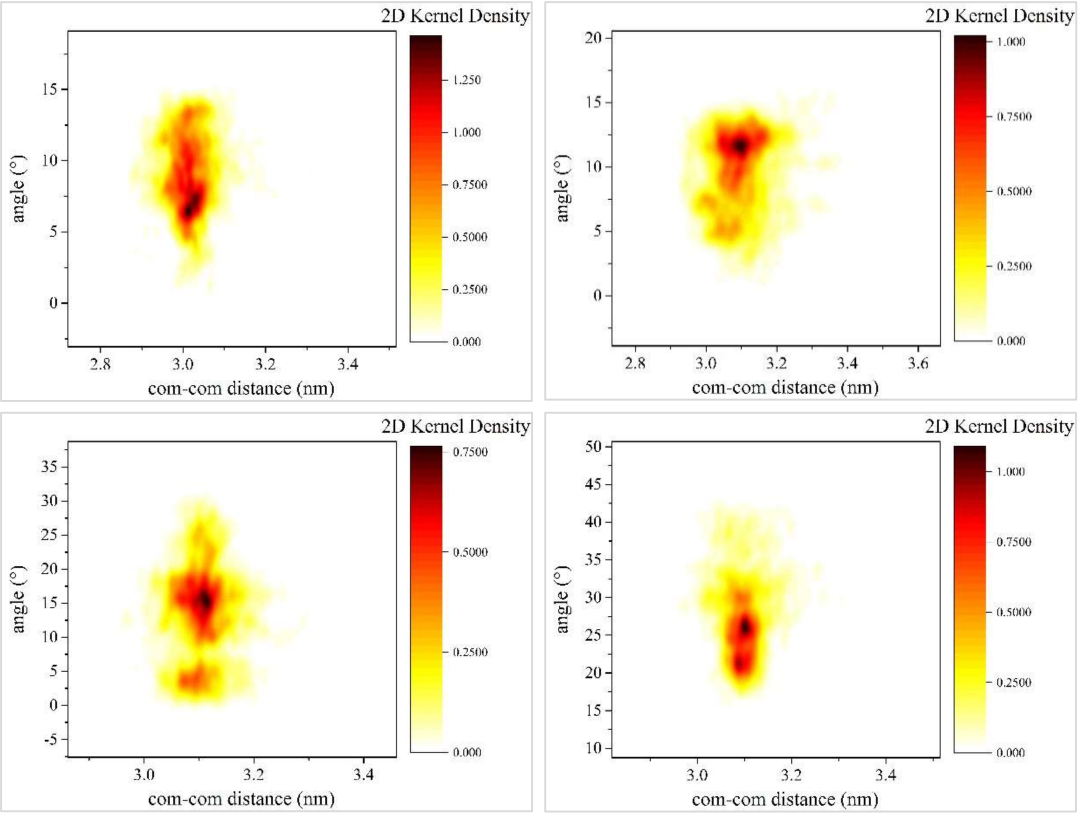

Figure S6. Probability density function of the average orientation angles between the eigenvectors of a pair of DNA molecules restrained at center-of-mass distances close to 3 nm: a) case of 65bp DNA minicircles in a 0.1M NaCl solution (system R65Na in Table 1), b) case of 65bp DNA minicircles in a 0.1M $CaCl_2$ solution (system R65Ca in Table 1), c) case of 24bp linear DNA fragments in a 0.1M NaCl (system L24Na in Table 1), d) case of 24bp linear DNA fragments in a 0.1M $CaCl_2$ solution (system L24Ca in Table 1). Eigenvectors corresponding to either the largest, or smallest eigenvalue of the respective gyration tensors are employed for the calculation in the case of linear DNA, or minicircle DNA, respectively.

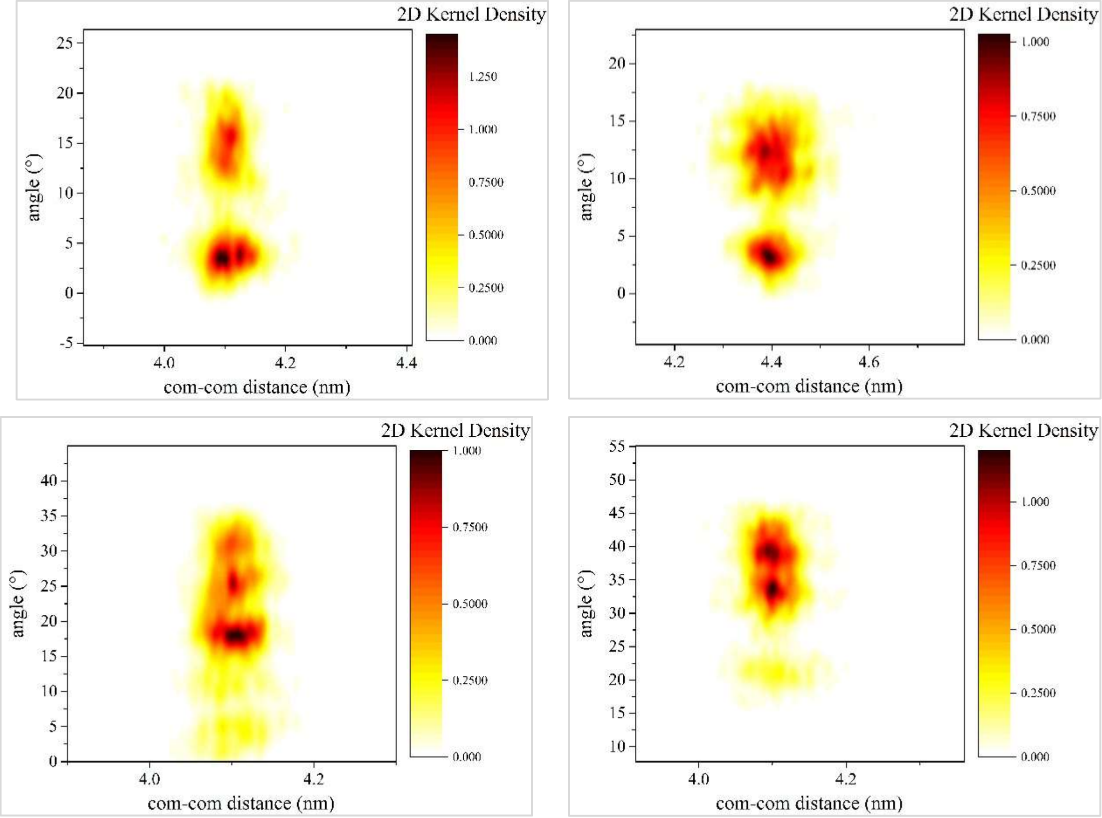

Figure S7. Probability density function of the average orientation angles between the eigenvectors of a pair of DNA molecules restrained at center-of-mass distances close to 4 nm: a) case of 65bp DNA minicircles in a 0.1M NaCl solution (system R65Na in Table 1), b) case of 65bp DNA minicircles in a 0.1M $CaCl_2$ solution (system R65Ca in Table 1), c) case of 24bp linear DNA fragments in a 0.1M NaCl (system L24Na in Table 1), d) case of 24bp linear DNA fragments in a 0.1M $CaCl_2$ solution (system L24Ca in Table 1). Eigenvectors corresponding to either the largest, or smallest eigenvalue of the respective gyration tensors are employed for the calculation in the case of linear DNA, or minicircle DNA, respectively.

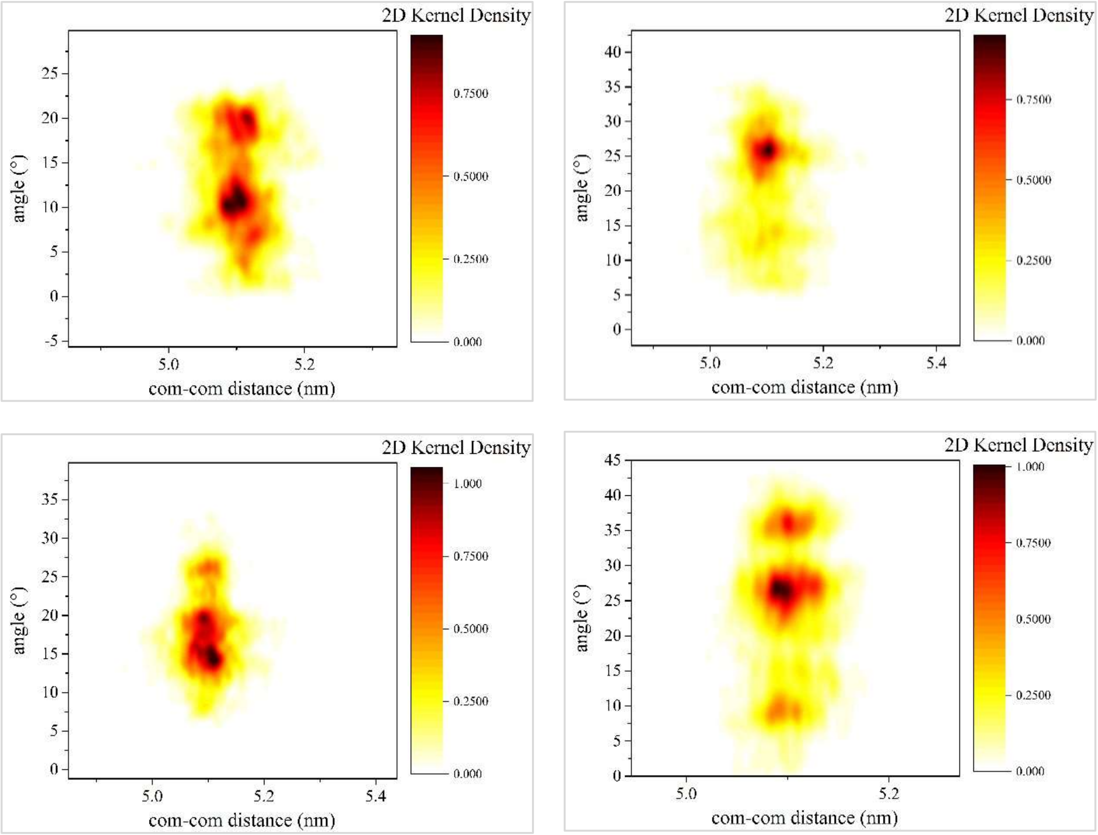

Figure S8. Probability density function of the average orientation angles between the eigenvectors of a pair of DNA molecules restrained at center-of-mass distances close to 5 nm: a) case of 65bp DNA minicircles in a 0.1M NaCl solution (system R65Na in Table 1), b) case of 65bp DNA minicircles in a 0.1M $CaCl_2$ solution (system R65Ca in Table 1), c) case of 24bp linear DNA fragments in a 0.1M NaCl (system L24Na in Table 1), d) case of 24bp linear DNA fragments in a 0.1M $CaCl_2$ solution (system L24Ca in Table 1). Eigenvectors corresponding to either the largest, or smallest eigenvalue of the respective gyration tensors are employed for the calculation in the case of linear DNA, or minicircle DNA, respectively.

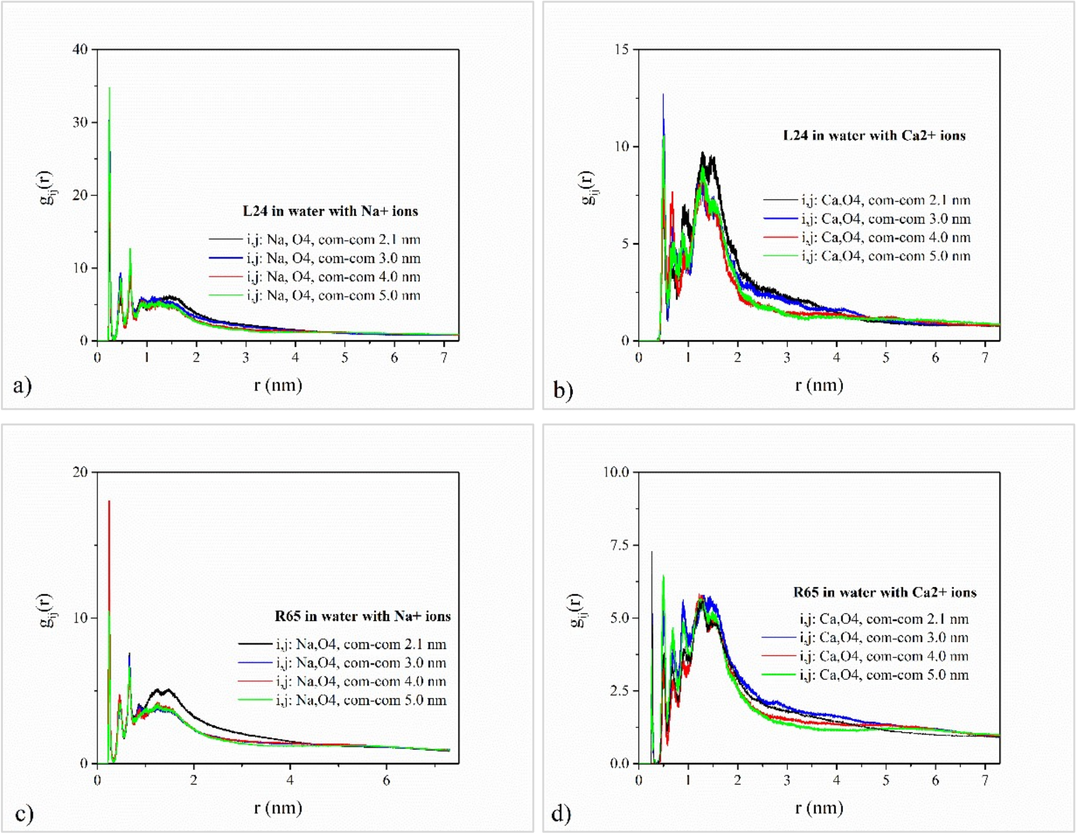

Figure S9. MD simulation results for the radial pair distribution function between salt counterions and major groove O4 oxygens and its dependence on DNA topology and counterion valency. Top row corresponds to the system of two linear DNA molecules with a) Na^{+} counterions, and b) Ca^{2+} counterions. Bottom row corresponds to the system of two DNA minicircles with c) Na^{+} counterions, and d) Ca^{2+} counterions.

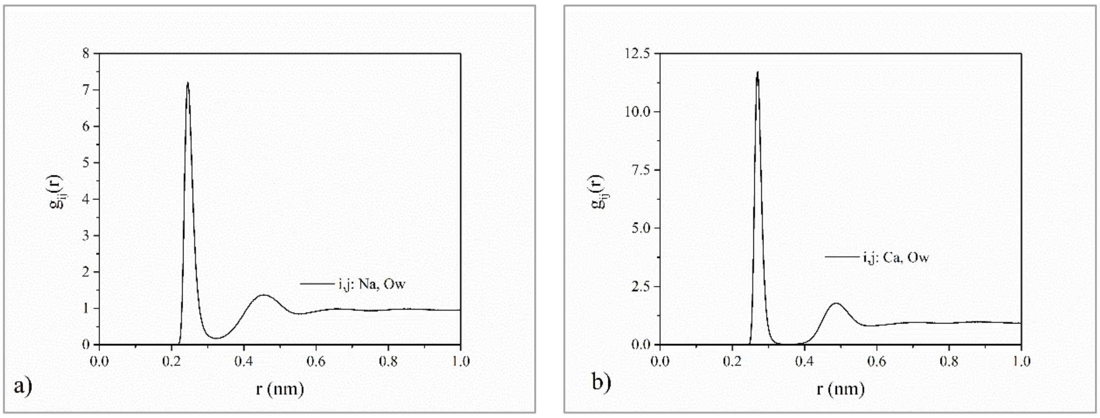

Figure S10. MD simulation results for the radial pair distribution function between salt counterions and water oxygen atoms Ow and its dependence on DNA topology and counterion valency. Results shown correspond to the system of two linear DNA molecules with a) Na^+ counterions, and b) Ca^{2+} counterions.

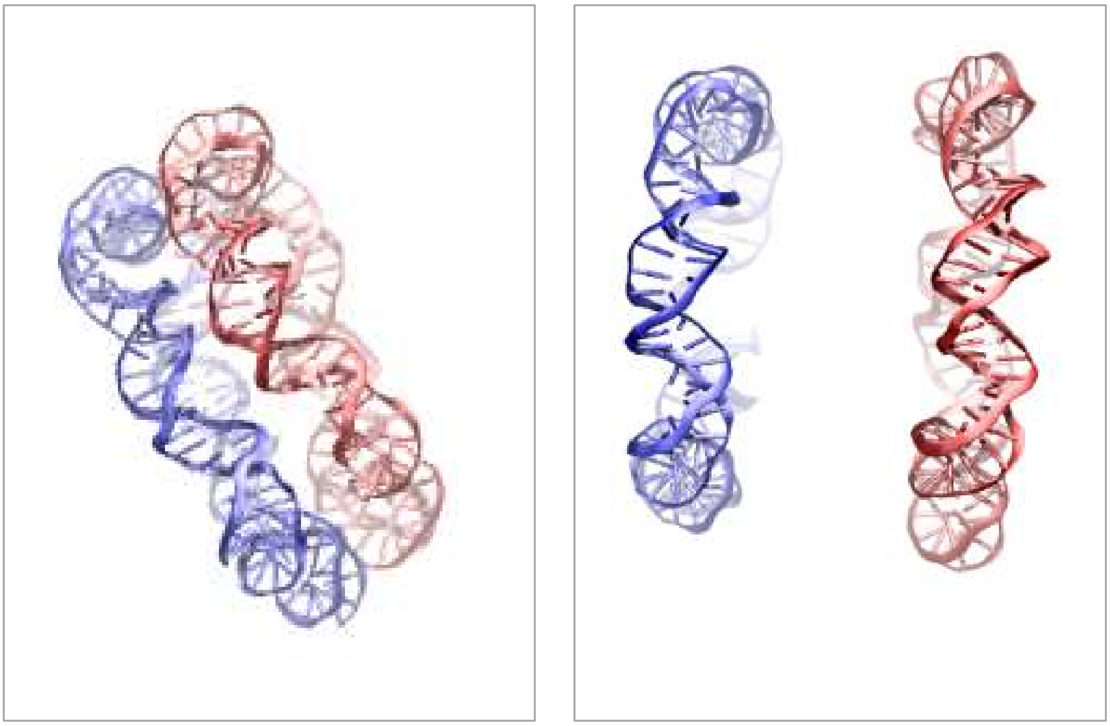

Figure S11. Typical atomistic snapshots of the instantaneous configuration of pairs of 65bp DNA minicircles (in a 0.1M CaCl2 solution) restrained at center-of-mass distances of: a) 2.0 nm and b) 5.0 nm. The ionic force field proposed by Mamatkulov, Fyta, and Netz (reference 53 in manuscript) has been employed for the conduction of the MD simulations.

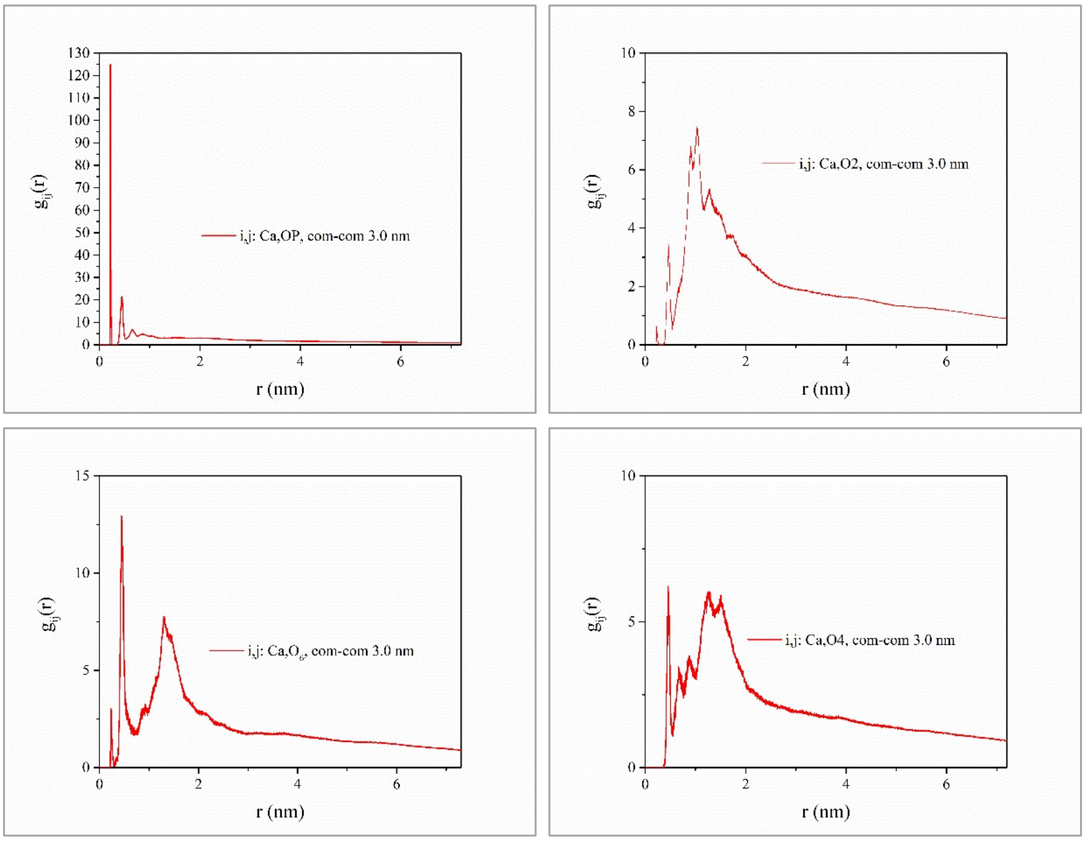

Figure S12. MD simulation results for the radial pair distribution function between calcium counterions and DNA oxygen atoms for the case of pairs of 65bp DNA minicircles (in a 0.1M CaCl2 solution). Top row corresponds to the case of phosphate group oxygens (results have been averaged for the O1P and O2P type atoms following the amber force field naming convention) and minor groove O2 oxygen atoms. Bottom row corresponds to the case of major groove O6 and O4 oxygen atoms. The ionic force field proposed by Mamatkulov, Fyta, and Netz (reference 53 in manuscript) has been employed for the conduction of the MD simulations.

System	Radius of 1st hydration shell (nm)	Radius of 2nd hydration shell (nm)	Hydration number in 1st hydration shell	Hydration number in 2nd hydration shell
L24Na	0.241	0.452	5.46	3.7
L24Ca	0.264	0.485	10.74	5.37

Table S1. Estimated radii of first and second hydration shells of sodium and calcium counterions, and respective hydration numbers in the first and second hydration shell, as estimated from MD simulations. Results shown for linear DNA fragments, systems L24Na and L24Ca.

	$R_{com-com}$= 2 nm	$R_{com-com}$= 3 nm	$R_{com-com}$= 4 nm	$R_{com-com}$= 5 nm
L24Na	0.20±0.04<br>0.46±0.12	0.23±0.08<br>0.19±0.05	0.22±0.06<br>0.21±0.04	0.23±0.06<br>0.26±0.06
L24Ca	0.22±0.05<br>0.26±0.01	0.35±0.06<br>0.20±0.05	0.20±0.04<br>0.21±0.05	0.20±0.05<br>0.21±0.05
R65Na	0.26±0.03<br>0.26±0.03	0.30±0.04<br>0.30±0.04	0.27±0.03<br>0.30±0.03	0.30±0.04<br>0.31±0.04
R65Ca	0.24±0.04<br>0.27±0.01	0.31±0.05<br>0.25±0.04	0.25±0.02<br>0.28±0.03	0.29±0.01<br>0.27±0.01

Table S2. RMSF estimates from MD simulations (units in nm): contour-wise mean values and standard deviations, estimated along the backbone of each DNA molecule. Values are shown in two rows for each system studied, each row corresponding to the respective molecule 1 and molecule 2, as labelled in Figures 5-6.
